# Alexander Fisher

**Published:** 1882-04

**Authors:** 


ALEXANDER FISHER, M.D., was born in Lancaster, Worcester county, Mass., August 12, 1804. After receiving an Academic education, he began the study of medicine in 1831, under the private instruction of Dr. Geo. W. Richards, of Camillus, N. Y. He entered the College of Physicians and Surgeons at Fairfield, Herkimer county, N. Y., from which institution he received the degree of Doctor of Medicine in 1834, and afterward practiced a short time in partnership with his preceptor, Dr. Richards.

In June, 1835, he removed to Western Star, Summit county, Ohio, where he was engaged in active practice until the fall of 1849. The winter of 1849–'50 he spent in Philadelphia in medical study; and in the spring of 1850 he resumed practice in Akron, Ohio. In 1854, failing health obliged him temporarily to suspend his professional work. Having regained his health, in the following year he removed to Chicago, where he continued faithfully to practice his profession until the infirmities of age compelled him—not long before his death—to relinquish its labors.

He was modest and unobtrusive in his manners, but gave to each recurring day as much patient labor as was necessary for the faithful performance of its duties. He dealt honestly with his patients; he had a nice sense of professional honor; in every walk of life he was a worthy citizen, and enjoyed the respect and esteem of all who knew him. Though engaged in general practice, he gave especial attention to surgery, and among his operations of interest may be mentioned a case of ligature of the external iliac artery, published in the *American Journal of the Medical Sciences*, April, 1856. He served for a term as a volunteer surgeon in the war of the rebellion. He was Emeritus Professor of Surgery in the Woman's Hospital Medical College, and a member of the Chicago Medical Society, and also of the Society of Physicians and Surgeons, of Chicago. He died Feb. 15, 1882. Though twice married, he died a widower, leaving two sons in business in Colorado.

THE Chicago Medical Society, at the meeting held on March 6, voted the following resolutions, and recommended their publication in the medical journals of the city:

WHEREAS, Alexander Fisher, M.D., an honorable, eminent and efficient Fellow of this Society, in the ripeness of his years, and after a long and successful practice of medicine and surgery, has been called to his reward in another stage of existence, where pain is unknown and the weary are at rest; be it therefore

*Resolved*, That whilst we recognize the fiat of our Heavenly Father, in removing our friend and Fellow from our midst, as the inevitable destiny of man, it is yet with sorrow we remember that we shall see his face no more, nor again listen to his voice in the councils of this society.

*Resolved*, That a committee of three be appointed by the President to present these resolutions to the family of the deceased Fellow, with expressions of our sympathy and sorrow.

DRS. JOHN BARTLETT,

G. C. PAOLI,

R. C. HAMILL,

D. W. GRAHAM, } Committee.

At the meting of March 20 the following committee was appointed to present the same resolutions to the family of the deceased: Drs. N. S. Davis, L. H. Montgomery and A. Reeves Jackson. E. I.

---

AT a special meeting of the Alumni Association of Cook County Hospital, held at the Grand Pacific Hotel, the following resolutions were passed with reference to the demise of Dr. D. S. Root, an old alumnus. Just as the Association was congratulating itself upon its integrity it became its painful duty to record its first death:

WHEREAS, It has pleased Providence to remove from our midst a beloved brother,

RESOLVED, That in the death of Dr. D. S. Root this Association, and the medical profession as well, have lost one who had gained an enviable position in his chosen calling.

RESOLVED, That in his life we recognize an example of great industry, unsullied integrity and excellent scholarship, which made his career of great service to his fellow-men and his death deeply regretted by all with whom he has been associated.

